# A Novel Supra-Brow Combined with Infra-Brow Lift Approach for Asian Women

**DOI:** 10.1007/s00266-016-0632-y

**Published:** 2016-03-22

**Authors:** Maoguo Shu, Lin He, Yingjun Su, Junli Shi, Xi Zhang, Xiangyu Liu, Xueyuan Yu

**Affiliations:** Institute of Plastic Surgery, Xijing Hospital, FMMU, Changle West Road 127#, Xi’an, 710032 Shaanxi China

**Keywords:** Eyebrows, Rhytidoplasty, Blepharoplasty, Suture techniques, Cicatrix

## Abstract

**Background:**

Direct brow lift surgery remains popular among Asian women despite its disadvantages. The traditional direct brow lift by a supra-brow incision is not suitable for Asian women because of their unique facial features, such as higher eyebrows, wider upper eyelids, and more orbital fat. Therefore, we designed a novel brow lift technique via a supra-brow combined with an infra-brow approach for Asian women.

**Methods:**

An area of skin above and below the eyebrow was measured, demarcated, and surgically removed. The redundant orbicularis oculi muscle (OOM) was excised while keeping the frontalis muscle intact. The OOM in the inferior flap was elevated and sutured to the frontalis muscle. In cases of puffy eyelids, orbital fat was partially removed through an infra-brow incision. Finally, a series of modifications were performed to reduce post-operative scarring.

**Results:**

A total of 496 patients underwent this surgery from July 2009 to December 2013 and 432 patients were followed up for at least 6 months after surgery. Post-operative scars, in most patients (428/432), were inconspicuous. There were no facial nerve injuries documented and eight patients reported transient forehead numbness. The height of the palpebral fissure was increased but there was no marked increase observed of the distance between the upper eyelid edge and the eyebrow. In follow-up visits, 409 out of 432 patients (94.7 %) were satisfied with their surgical results.

**Conclusions:**

This new brow lift technique via a supra-brow combined with an infra-brow approach provided a simple and safe surgical repair of lateral brow ptosis, upper eyelids hooding, and crows’ feet in Asian women. The surgical outcomes were predictable and the scars were inconspicuous.

**Level of Evidence IV:**

This journal requires that authors assign a level of evidence to each article. For a full description of these Evidence-Based Medicine ratings, please refer to the Table of Contents or the online Instructions to Authors www.springer.com/00266.

## Introduction

Eyebrows are important facial features, as people tend to focus on the periocular region while engaging socially. The eyebrow curves upward and peaks laterally at one-third of its distance. Its natural position is at or slightly higher than the supraorbital margin [1]. Eyebrow ptosis, a characteristic among the first manifestations of aging, usually accompanies upper eyelid hooding and crow’s feet. Eyebrow lift surgery is corrective, as it projects youth and beauty.

The purpose of a brow lift is to adjust the eyebrow at an esthetically appropriate position. There are many kinds of brow lift methods today. A direct brow lift by supra-brow approach is a common method, as it is easy, effective, and minimally invasive, but scarring after surgery is a limitation. Despite this technique becoming less popular and being replaced by the endoscopic brow lift, it is still popular among Asian women because of progress in scar treatment and the widely used eyebrow tattoo technique to camouflage scars.

Compared with Caucasian women, Asian women have higher eyebrows, wider upper eyelids, and more orbital fat [2]. In many aging Asian women, upper eyelid hooding, and swelling are more concerning than eyebrow ptosis itself. In these patients, the traditional direct brow lift via the supra-brow approach may raise the eyebrow higher than normal and broaden the upper eyelid width, which can result in an expression of surprise. Therefore, we designed a new eyebrow lift technique via a supra-brow combined with an infra-brow approach in Asian women. Additionally, modifications were made to reduce post-operative scarring. Surgical outcomes were predictable and the scars were inconspicuous in most patients.

## Patients and Methods

### Patients

A total of 496 female patients who were considered suitable for brow lift surgery underwent the procedure from July 2009 to December 2013. Patients ranged from 38 to 70 years old; most were 40–55 years old, with a mean age of 48 ± 8 years old.

### Pre-operative Measurement

A frontal view photograph of each patient was taken prior to surgery. The symmetry of the eyebrow was analyzed and recorded. The height of the palpebral fissure (HPF), the distance between the upper eyelid margin and the eyebrow (DEE), and the distance between the eyebrow and the hairline (DEH) were measured before the surgery [3]. The DEE and DEH were measured at the vertical line of the eyebrow peak point.

**Fig. 1:**
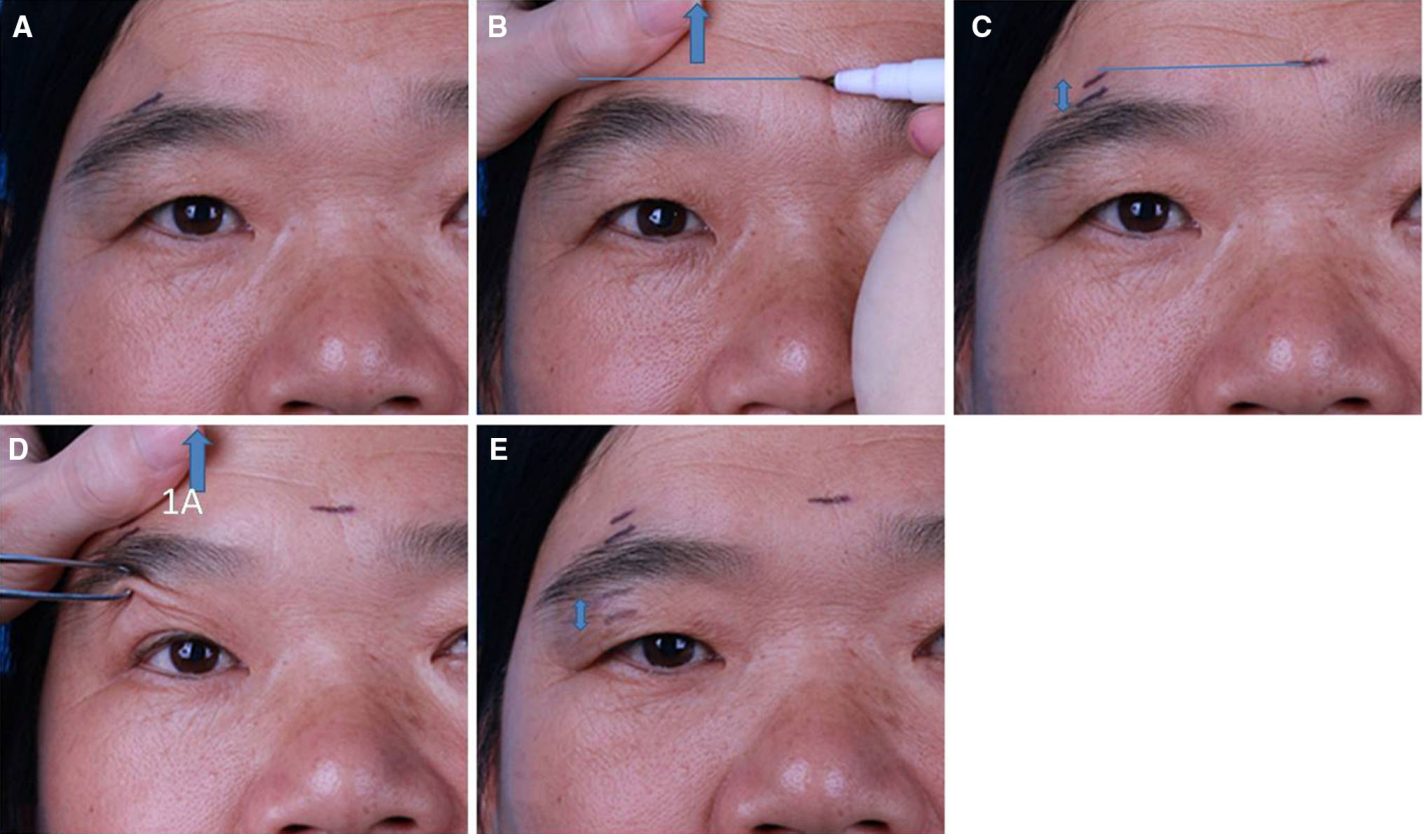
Upper eyebrow pre-operative measurements. **a** *Mark* on the pre-operative eyebrow peak point. **b** Identify the new eyebrow peak point level when the eyebrow is pushed up. The *blue arrow* indicates the raise of the eyebrow. **c** The distance between the *two marks* of eyebrow peak points defines the width of the skin to be excised. **d** With the eyebrow pushed up, the excess upper eyelid skin is clamped by wide smooth forceps. **e** The two points on the upper eyelid skin clamped by the clamp tips are marked along the *vertical line* through the eyebrow peak point and the distance between the two points would be the width of the skin to be excised

With the patient in a seated position and relaxed, a point was marked on the peak point of the eyebrow (Fig. 1a). Then, the eyebrow was elevated manually to the desired position and the new level of the eyebrow peak point was marked (Fig. 1b). The distance between the two points is the width of the skin area to be excised above the eyebrow (Fig. 1c). Next, with the eyebrow elevated to the designed location by an assistant, the excess upper eyelid skin below the eyebrow was clamped by wide smooth forceps. After the amount of clamped skin and tissue was adjusted and the ideal HPF and DEE were met, the two clamped points on the upper eyelid skin were marked at the vertical line through the eyebrow peak point (Fig. 1d, e). The distance between the two points would be the width of the skin area to be excised below the eyebrow (Fig. 1e).

### Operative Procedure

**Fig. 2:**
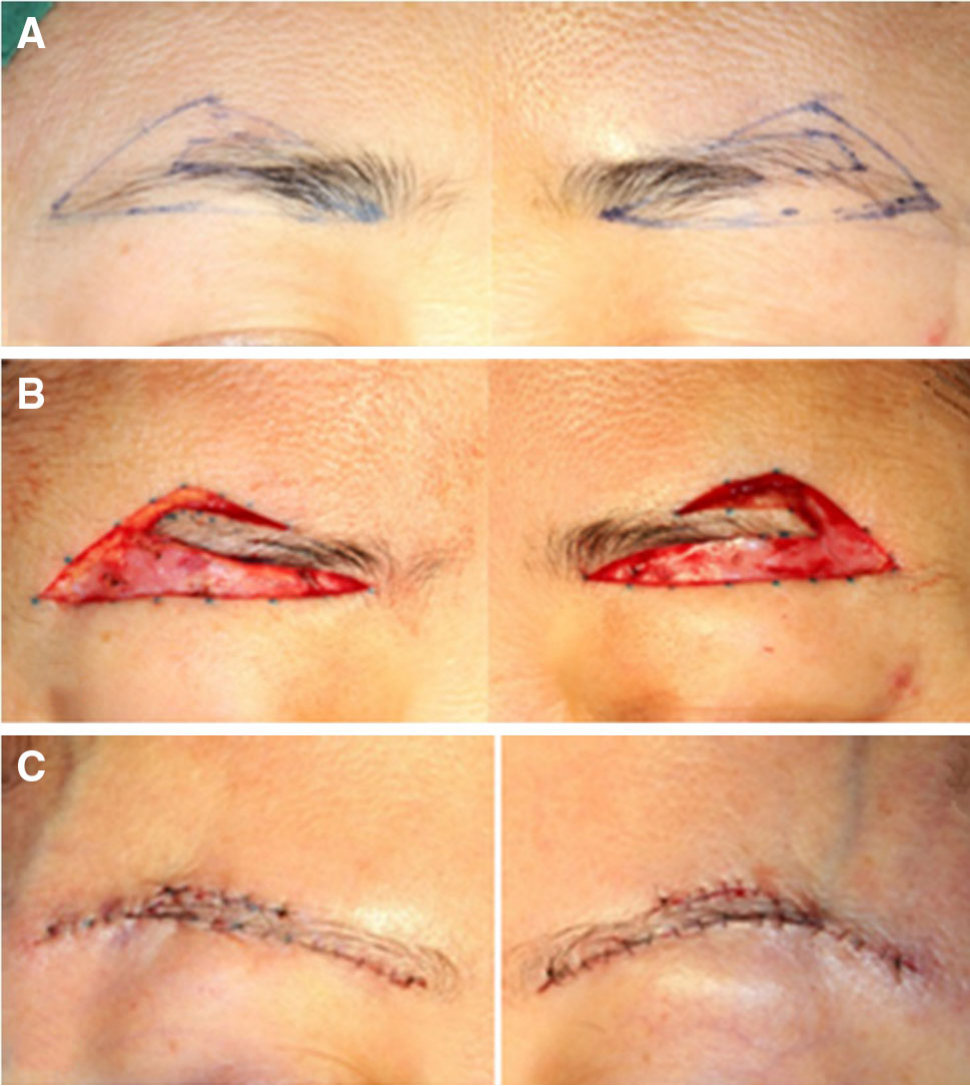
Surgical design and operation procedure. **a** The *marked areas* of skin to be excised show a swallow-tail shape. **b** Wound areas after the skin and soft tissue are excised. **c** The shape of brow after suture

In the supine position, after adequate skin preparation and draping, the incision lines were marked according to pre-operative measurements and assessment (Fig. 2a). The excised skin is swallow-tail shaped. The line could be within the eyebrow and the end part of the eyebrow could be excised accordingly. The upper line began at the supraorbital nerve notch (SON) and the lower line began at the beginning of the eyebrow. Both lines ended at the tip of the eyebrow.

After adequate infiltration anesthesia was administrated (5 ml of 2 % lidocaine, 5 ml of 0.75 % bupivacaine and 0.1 ml of 0.1 % adrenaline, 3–5 ml per side), incisions were performed along the marked lines and deep to fat tissue. To avoid injury to the eyebrow hair follicles, the incisions close to the eyebrow were made parallel to hair follicles. The skin and fat tissue were dissected and removed above the muscle layer by eye scissors from the lateral end to the nasal end. The dissection led to minimal bleeding due to locally applied adrenaline solution (Fig. 2b).

The margins of the incisions were dissected subcutaneously to facilitate suturing. The upper eyelid flap was dissected at ~1 cm, and the other incisal margins were dissected at ~3 mm. The frontalis in the supra-brow area was not excised, so the emotional expression of the eyebrow would not be affected. The redundant orbicularis oculi muscle (OOM) below the eyebrow was excised, and careful dissection was performed at the SON area to avoid injuring the supraorbital nerve. In some cases with severe glabellar wrinkles, the corrugator and procerus muscles were damaged carefully under direct vision. Additionally, in some cases of puffy eyelids, orbital fat was removed in part via an infra-brow incision.

After carefully achieving hemostasis, the eyebrow was fixed at an appropriate position via suturing it to the periosteum with 5-0 absorbed sutures. The OOM in the inferior flap was subsequently pulled up and fixed to the frontalis muscle with 5-0 absorbable sutures. Care was taken as to not have the sutures be tight, as in case of upper eyelid dysraphism. After a desired eyebrow and upper eyelid position were obtained, the wound was closed. The subcutaneous layer was closed by modified fully buried vertical mattress with 5-0 sutures. This method proved reliable for closing of wounds with tension [4]. The skin layer was closed by continuous stitches with 7-0 nylon sutures (Fig. 2c).

### Statistics

Statistical analysis was performed using SPSS 23.0 for Windows. A paired *t*-test was used to examine the differences in HPF, DEE, and DEH between the pre-operative and post-operative photos. All statistical tests were based on two-tailed probability and the criterion for significance was set at *P* < 0.05.

## Results

**Fig. 3:**
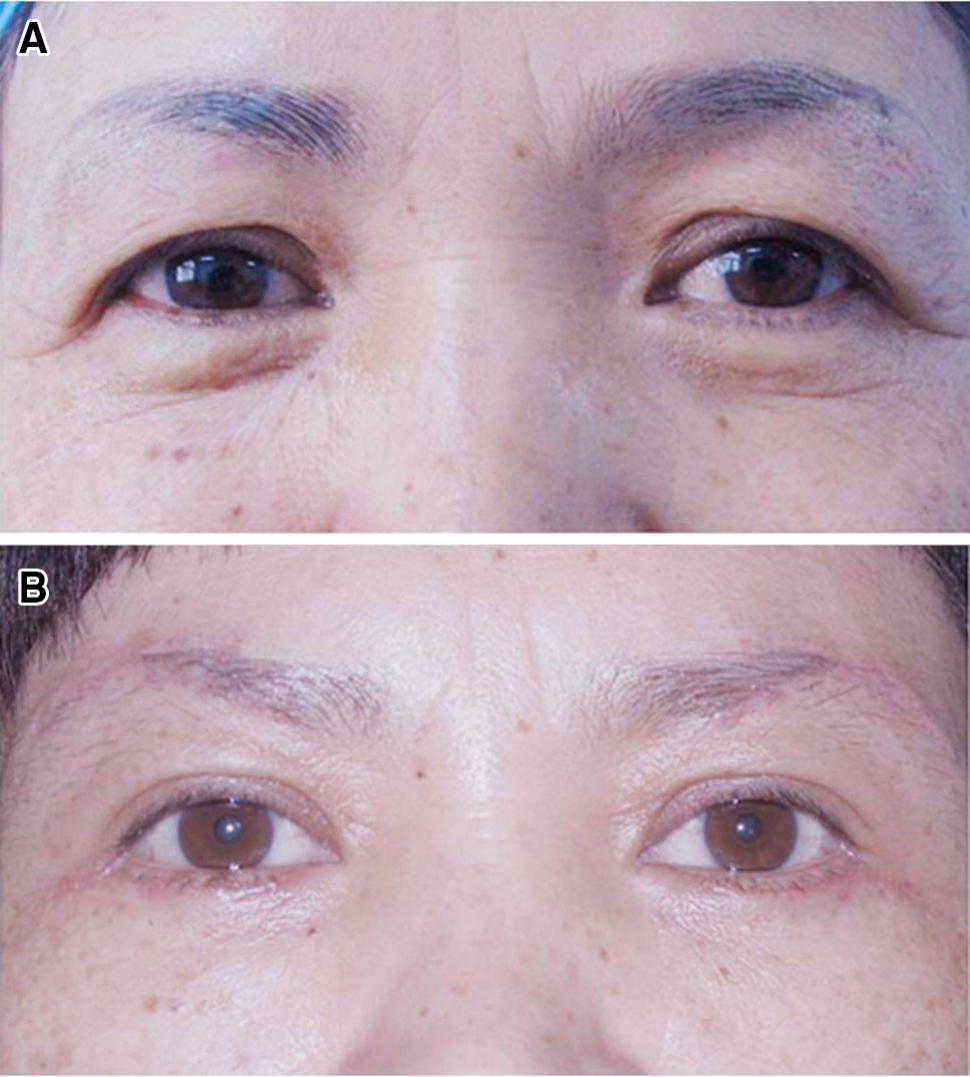
The effect of surgery. **a** Pre-operation: a 48-year-old women with upper eyelid hooding and crows’ feet. **b** 6 months after operation: the lateral upper eyelid hooding and crows’ feet were improved and the scars were inconspicuous

**Fig. 4:**
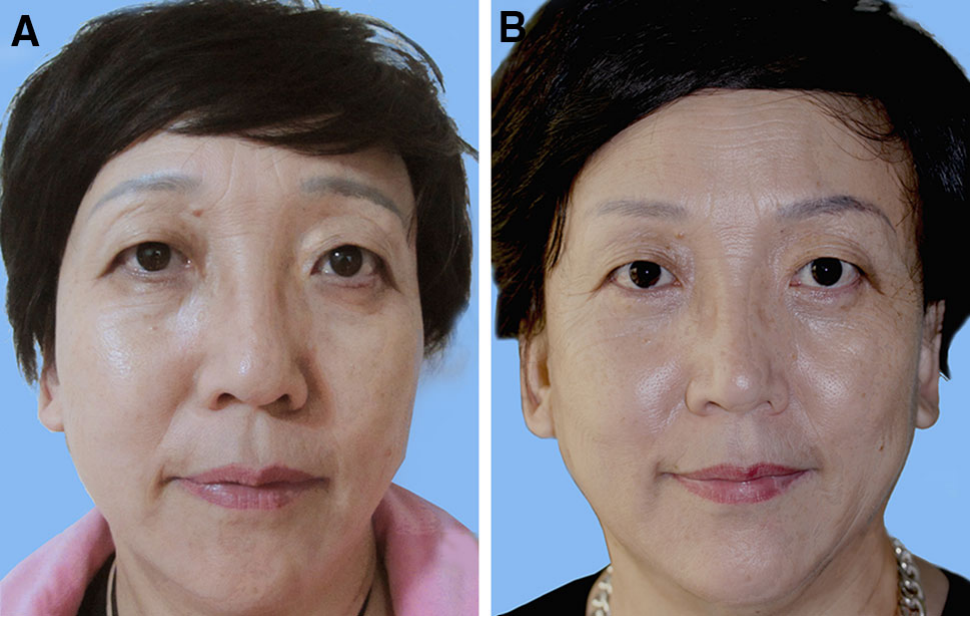
The effect of surgery. **a** Pre-operation: a 52-year-old women with lateral brow ptosis, upper eyelid hooding and crows’ feet. **b** 4 years after operation

A total of 496 female patients underwent this procedure from July 2009 to December 2013; 432 patients were followed for at least 6 months and participated in an outcomes evaluation. The follow-up period was 6 months to 4 years, with an average of 12 months. The skin suture was removed at day 6 and the tissue swelling at the surgical sites subsided at 1–2 weeks after the operation. The lateral brow ptosis, upper eyelids hooding, crows’ feet were notably improved and the scars were inconspicuous after 6 months post-operatively (Fig. 3a, b). Patients’ positive outcomes were noted for up to 4 years after surgery (Fig. 4a, b). The initial surgical scar could be camouflaged by an eyebrow tattoo or eyebrow pencil. Most patients experienced no discomfort during their daily activities, even at the early recovery phase (between 1 and 2 months post-operatively).

Intraoperative complications such as supraorbital nerve/supratrochlear nerve injuries, obvious scars, and asymmetry were observed in this patient cohort. The outcomes are shown in Table 1. No facial nerve injuries were observed. Forehead numbness occurred in eight patients, which gradually subsided within 6 months. Five patients with asymmetric eyebrows and seven patients who were unsatisfied with the effect of surgery had a secondary operation after 6 months. There were four patients with obvious scars ineffective to laser and silicone gel treatments. The scars were ultimately camouflaged by eyebrow tattoos.

**Table 1:** Complications in 432 Chinese females after brow lift

	Facial nerve injury	Supraorbital/supratrochlear nerve injury	Obvious scar	Asymmetry	Inefficient lift	Total
Cases	0	8	4	5	7	24
%	0	1.9	0.9	1.2	1.6	5.6

To analyze the effects of surgery, the HPF, DEE, and DEH were measured via digital images (full 1:1 standardized horizontal plane photographs) obtained before and 6 months after the operation. As shown in Table 2, the HPF was increased significantly and the DEH was decreased significantly after the operation. Furthermore, no marked change was observed on the DEE after the operation.

**Table 2:** Changes in HPF, DEE, and DEH of 432 Chinese female after brow lift

Parameter	Pre-operation	Post-operation	Difference	% (range)	*P* value
HPF	8.1 ± 0.5	8.5 ± 0.5	+0.3 ± 0.2	+4.3 ± 2.6	0.000^*^
DEE	17.9 ± 1.0	17.8 ± 1.2	−0.1 ± 0.4	−0.4 ± 2.2	0.220^#^
DEH	60.3 ± 5.5	55.4 ± 4.9	−3.4 ± 1.3	−5.6 ± 2.1	0.000^*^

Data are shown as mean of distance (mm) measured from digital images

^*^Significant differences between pre- and post-operation (paired *t*-test, *P* < 0.05)

^#^No significant differences between pre- and post-operation (paired *t*-test, *P* > 0.05)

We also asked the patients to evaluate surgical outcomes at 6 months after surgery based on five criteria: (1) the effect of the elevation of lateral brow and upper eyelids, (2) modification of crow’s feet, (3) symmetry, (4) scarring, and (5) overall appearance. In general, 94.7 % patients were satisfied with surgical outcomes.

## Discussion

The brow lift technique was first described by Passot [5] nearly 100 years ago. He proposed a method of excising excess skin and fat tissue by a supra-brow incision to elevate the eyebrow and diminish crow’s feet. Despite the procedure’s ease and efficacy, it had become less popular because of visible scarring. Later, coronal incision approach brow lift surgery became widely used as well as upper face rejuvenation, but these methods have also become rare because of the potential for extensive surgical scarring, alopecia, and operative trauma [6]. The endoscopic technique brought a revolution in brow lift and has been popular in the recent 20 years. But, to achieve long-term effects, eyebrow fixation by special materials was needed in endoscopic brow lift [7]. The expensive equipment and materials restricted endoscopic technique use. There are surgeons that argue that they can do brow lifts with small incisions as performed in endoscopic procedures without endoscopy [8], but procedures as such require a high degree of experience.

Besides a hairline approach, the indirect brow lift via blepharoplasty in which the eyebrow is elevated and fixed to the periosteum by suture through a supratarsal incision is also reported by surgeons [9]. In this technique, it is difficult to achieve adequate eyebrow elevation and the fixed point may be dimpled. In addition, half of Asian women do not have a supratarsal crease nor do they desire an artificial supratarsal crease.

Although a direct brow lift by the supra-brow approach can leave visible scars after surgery, the scars can be minimized with careful wound closure and post-operative anti-scar therapy. Booth et al. [10] reported the use of the direct brow lift to treat brow ptosis and achieved good outcomes and high levels of patient satisfaction. The scars could be acceptable by patients through careful wound closure. Lee et al. [11] also reported using the modified direct brow lift with a focus on reducing post-operative scarring in Korean patients with successful outcomes. So, direct brow lift is still one of the choices for aging people who want to look younger.

The position of the eyebrows on aging faces can vary notably. Some reports indicate that the eyebrow’s level becomes lower with age, but this change was not observed by other investigators [12]. From our experience, Asian women who seek eyebrow lift surgery mostly are between the ages of 40–60 years old. The prominent facial features of aging in this patient group are lateral brow ptosis and upper eyelids hooding, yet the position of the inner one-third of the eyebrow remains stable.

Moreover, the peri-orbital characteristics of Asian women are different from Caucasian women. Asian women have a larger palpebral fissure height, a steeper angle of the ocular axis, higher eyebrows, and wider upper eyelids as compared to those of Caucasian women. The average distance of the midtarsal to the lower end of the brow in Asian women is 11.6 ± 2.7 mm, significantly wider than 7.8 mm ± 2.0 mm in Caucasian women. But the average distance of the lateral end of the brow to the lateral canthus has no significant difference [2]. In addition, Asian women have more orbital and subcutaneous fat. So, in older Asian women, lateral brow ptosis and upper eyelid hooding look more serious than it actually is. The goal is to raise the lateral brow and resolve the upper eyelid drooping without broadening the upper eyelid width. These ethnic characteristics make many Asian women poor candidates for traditional direct brow lift by a supra-brow approach.

To solve the eyebrow ptosis and upper eyelid hooding for those who are not candidates for traditional direct brow lift, we designed a new eyebrow lift technique via a supra-brow combined with an infra-brow approach. This new method combined the advantages of a direct brow lift and the subbrow blepharoplasty technique [13]. We raised the drooping eyebrow and fixed it at an appropriate position through a supra-brow incision, and then made an infra-brow incision to manage upper eyelid hooding and swelling. The excess upper eyelid skin, muscle, and orbital fat are removed by an infra-brow incision and the upper eyelid is raised by suture.

Besides optimally treating eyebrow ptosis and upper eyelid hooding, this technique boasts other advantages. By adjusting incision lines, the shape of the eyebrow can be changed altogether. Glabellar wrinkles can be diminished by incising the corrugator and procerus muscles through infra-brow incisions, but it needs to be stressed that careful surgical technique must be done at the SON and glabellar area to avoid injury to the supraorbital and supratrochlear nerves.

The reduction of post-operative scarring is another aim of our study. The visible scars after surgery are unacceptable to patients. To prevent scarring during the procedure, it is imperative that the scalpel is parallel to hair follicles to allow regrowth of hair through the incision. The edge of the incision is to be dissected from subcutaneous tissue to facilitate subcutaneous suturing, and the OOM should be sutured to the frontalis muscle to reduce wound tension. During our procedures, subcutaneous layers were sutured with a modified vertical mattress suture fully buried method, which reduced the tension of wounds and alleviated foreign matter-induced immune reactions in wound tissues [4]. Application of silicone gel was recommended to patients to treat post-operative scars after sutures were removed. In our patient cohort, most scars were inconspicuous 6 months after the operation.

Compared with traditional a direct brow lift, the eyebrow was fixed and the orbicularis oculi was elevated and sutured to frontalis, so effects are long lasting. In our follow-up, many patients were pleased with their results and appearance for more than 3 years. Longer effects of all patients need further observation.

This technique may not be suitable for most Asian men who have full eyebrows and narrow upper eyelids because the technique involves reshaping the eyebrow and thus the distance is shortened between the eyebrow and the eye. In cases of men who want an eyebrow lift, the traditional brow lift method is used.

## Conclusion

The direct brow lift is easy, effective, and minimally invasive. Although this technique has complications such as visible scarring and forehead numbness, these complications can be overcome by technique. The characteristics of Asian women who have higher eyebrows and wider upper eyelids as compared to those of Caucasian women make many of them poor candidates for a traditional direct brow lift via the supra-brow approach. We reported our experience with our new brow lift technique via a supra-brow combined with an infra-brow approach in Asian women, and also the experience on reducing post-operative scarring. The surgical outcomes were predictable and the scars mostly inconspicuous.
